# Community physicians’ attitudes towards meetings with representatives of pharmaceutical companies: a pilot study

**DOI:** 10.1186/s40545-023-00521-8

**Published:** 2023-01-25

**Authors:** Amit Koplovitz, Tamar Freud, Roni Peleg

**Affiliations:** 1 Faculty of Health Sciences, Ben-Gurion University of the Negev, Beer-Sheva, Israel; 2 Department of Family Medicine, Siaal Research Center for Family Medicine and Primary Care, Faculty of Health Sciences, Ben-Gurion University of the Negev, POB 653, 84105 Beer-Sheva, Israel; 3 Family Medicine and Primary Care Clinic, Clalit Health Services, Southern District, Beer-Sheva, Israel

**Keywords:** Community physicians, Pharmaceutical companies, Representatives of pharmaceutical companies, Conflict of interest, Marketing

## Abstract

**Background:**

Marketing by pharmaceutical companies has become an increasingly controversial issue for the medical community and the public. This controversy stems from the potential influence that pharmaceutical companies can wield through marketing on the medical community. This study assesses community physicians’ attitudes towards pharmaceutical companies and their representatives to get a better understanding of how their activities affect daily work in community clinics.

**Methods:**

A cross-sectional anonymous questionnaire-based study of 170 community physicians in southern Israel was conducted via convenience sampling. The questionnaire was designed to assess physicians’ attitudes about the nature of their relationships with representatives of pharmaceutical companies and possible associations with physicians’ demographic and professional profiles. The questionnaire was distributed, at weekly staff meetings in the study clinics, to a convenience sample of physicians, who agreed to participate in the study.

**Results:**

Most physicians did not have an extreme attitude on interactions with representatives of pharmaceutical companies. Interestingly, while they thought that pharmaceutical companies play an important role in medical progress, they did express concern regarding the risk of misleading information. While they believed that interactions between physicians and representatives of pharmaceutical companies had a negative effect on the clinic workflow, they were not in favor of prohibiting such interactions. Physicians who graduated from medical schools in Israel held a less sympathetic position towards these interactions.

**Conclusion:**

The anticipated heterogeneous attitudes of community-based physicians on interactions with representatives of pharmaceutical companies reflect an inherent complex relationship, with aspects that are specific to the Israeli medical field. Interestingly, physicians trained in other countries than Israel also have divergent attitudes, further affecting the socio-cultural impact on practitioner’s attitudes towards this intricate and often politicized topic. Open professional dialogue and targeted educational programs on the physician–pharmaceutical relationship, with more explicit regulation, could potentially ease the discomfort experienced by physicians, especially in the Israeli context and result in a clearer framework of interaction that would leverage the potential advantages while accounting for ethical and regulatory pitfalls.

**Supplementary Information:**

The online version contains supplementary material available at 10.1186/s40545-023-00521-8.

## Background

The main goals of new drug development are to prevent or reduce mortality and morbidity and improve quality of life. Drug development is a complex, multi-phased process that begins with the identification of a substance with therapeutic potential and confirmation of its suitability as a drug. The objective of this process is to determine the drug’s components, dosage, and possible routes of administration. Prior to clinical trials (i.e., administration in human subjects), the pharmacology and biochemistry of the drug are determined using an extensive range of in vitro and in vivo testing procedures, followed by several phases of clinical trials. Each phase is designed to answer a different research question and to review and verify information procured in the previous phase including assessment of a safe dose range, possible adverse effects, and the drug’s efficacy.

The next step is to compare the new drug to existing accepted treatment, if available, and the final step is to apply to a regulatory agency, e.g., the US Food and Drug Administration, to confirm that the new drug is safe and effective for use and can be registered with the pharmaceutical authorities for a marketing license. Once the drug is marketed, the pharmaceutical company and the regulatory agencies continue to implement ongoing regulation and oversight to assess efficacy and identify risks, including additional morbidity that might arise from long-term use [1]. Another goal of this process is to identify new indications for the drug, or other patient populations that could benefit from it, such as children.

This process requires a major investment of resources, including a significant amount of capital over a long period of time. These resources, which are required throughout all phases of drug research and development, usually come from pharmaceutical companies [2]. New drugs are patented for a limited time of about 17 years, beginning with patent registration, granting the patent holder a limited time frame to make significant profits without competition. After the patent expires, other companies are legally allowed to manufacture and market the drug [3, 4].

All these considerations underpin the tremendous investment of resources made by pharmaceutical companies in advertising and marketing [5]. In the USA and Canada, more money is spent every year for the promotion and sales of drugs than on drug development. These costs are directed at the public and the medical community through various media outlets and are manifested by the efforts of pharmaceutical representatives who market drugs directly and in person to physicians and other medical personnel in hospitals and community clinics. Physicians are a major target group for pharmaceutical companies through marketing representatives [6, 7].

The dynamic relationship between clinicians and the pharmaceutical industry, which is critical for the advancement and betterment of healthcare, has received attention and scrutiny in recent years. Pharmaceutical marketing has become an increasingly controversial issue in recent years in the general population and the medical community [8]. The controversy stems from the potential influence pharmaceutical companies can have on physicians and the medical community when marketing and advertising drugs. Aggressive pharmaceutical marketing often includes meetings between representatives of pharmaceutical companies and physicians, distribution of gifts, supplying drug samples, providing funding for conferences, and even the funding of flights and accommodations for overseas conferences sponsored by the company they represent [9, 10]. In the USA, giving gifts such as pens and mugs, and providing benefits such as fancy meals, show tickets, etc., are prohibited [11]. In May 2015, a three-part series published in the *New England Journal of Medicine* concluded that the negative impact of these conflicts has been overstated, even noting that large healthcare organizations in the world encourage interactions between physicians and the pharmaceutical industry for the benefit of patients [12–14].

However, representatives of pharmaceutical companies often contact physicians and other allied health providers while they are treating patients. These encounters can disrupt care and reduce the time available for physician–patient meetings [15]. These concerns have become so prominent in the Israeli context that in October 2018, the CEO of the Israeli Ministry of Health issued a directive on meetings for commercial purposes at Israeli health institutions. This directive restricted relationships between physicians and pharmaceutical companies to prevent conflicts of interest that could arise from business interactions with pharmaceutical companies and bias the physician’s professional judgment [16].

The pharmaceutical–clinician relationship is inherently complex, with a potential to provide tremendous benefit to the public, but also to cause it harm. As such, it should be studied, analyzed, and properly cultivated. To this end, we aimed to assess the attitudes of community physicians towards pharmaceutical companies and their representatives, and how these activities affect physicians, physician–patient encounters, and daily work in community clinics.

## Methods

This cross-sectional study was conducted during October 2019–October 2020.

### Study population

The study population included physicians (residents and specialists) who worked in Clalit Health Services’ community clinics in southern Israel. These clinics employ 373 family physicians (169 specialists and 204 residents) and 270 consultants. Physicians who refused to participate in the study were excluded.

### Sample size

The sample size calculation was based on the hypothesis that 45% of family physicians and 80% of consulting physicians would oppose meetings with representatives of pharmaceutical companies during office hours. In addition, we hypothesized that 50% of specialists in family medicine and 75% of residents in family medicine would hold the same position.

The sample size calculation was based on a 95% significance level with a power of 80%. The resulting sample size was 167 physicians divided into several subgroups with a ratio of family physicians to consultants of 1:1.4 and a ratio of residents to specialists of 1:2.

### Data collection methods and study tool

Since we could not find a dedicated questionnaire, with validated measurement tools, designed to explore the attitudes of physicians towards meetings with representatives of pharmaceutical companies, we developed a self-administered, customized, structured questionnaire, which was pre-tested in a pilot study to assess comprehensibility and clarity. In constructing the questionnaire, we aimed to examine five main axes: (1) physicians’ level of trust in pharmaceutical-provided information and their view on its reliability; (2) checks and balances that practitioners apply to such information, e.g., researching it independently; (3) pharmaceutical-provided information as an incentive for acquiring up-to-date knowledge; (4) willingness to receive different forms of compensation from pharmaceuticals; and (5) the feasibility of such interactions within the intensive workflow of Israeli physicians. The questionnaire was designed in a Likert-scale format (the full questionnaire is shown in Additional file 1: Table S1). The pre-study pilot included two parts: in the first physicians completed the questionnaire and in the second, they were requested to provide feedback as to clarity of the questionnaire, and whether any other content was needed.

The final questionnaire had two distinct sections:

- The first evaluated physicians’ attitudes towards the nature of their relationships with representatives of pharmaceutical companies.
- The second related to the physician’s demographic and professional profile.

The next phase was to distribute the anonymous questionnaire, by convenience sampling, to physicians, who agreed to participate in the study. This was done at weekly staff meetings in the study clinics. The questionnaire was short, succinct, and carried the university's insignia, to increase participation [17]. The same researcher administered the questionnaire to enhance consistency in response behavior among the physicians.

### Ethical aspects

All methods were carried out in accordance with relevant guidelines and regulations. The study protocol was approved by the Ethical Committee of the Faculty of Health Sciences, Ben-Gurion University of the Negev, Beer-Sheva, Israel, prior to commencement of the study. That committee waived the study from the need to sign informed consent forms, according to the criteria used by the committee to establish whether informed consent forms are to be used, because the study questionnaires are given to physicians. The Internal Review Board-Helsinki committee of the Meir Medical Center Kfar-Saba granted an exemption on May 14th, 2019.

### Statistical analysis

Data analysis was performed using the SPSS program, version 25.

Comparisons were conducted between physicians in community clinics with consultants, between residents and specialists, between male and female physicians, and between physicians who graduated from medical school in Israel or abroad. Differences in categorical variables were calculated using Chi-square or Fisher exact tests according to the number of respondents. Differences in continuous variables were calculated by one-way ANOVA or *t*-test, as appropriate.

An overall attitude score was calculated as the mean score for all 11 questions. Lower scores indicated more negative attitudes. For this calculation the score was reversed in questions 4, 5, 8, and 11 (1 = yes, very much so, 5 = definitely no).

A linear regression model was used to determine factors associated with negative attitudes towards encounters with representatives of pharmaceutical companies. *p* < 0.05 was considered statistically significant in all tests.

## Results

### Response rate

One hundred and eighty-seven physicians received questionnaires and 170 physicians completed them (90.9% response rate). The main reason for not responding was a busy work schedule.

### Physicians’ characteristics

Seventy-eight (45.9%) of the 170 physicians were women, 139 (81.7%) were family physicians and 31 (18.3%) were consultants. The mean age was 44.6 ± 12.5 years. Socio-demographic characteristics and other relevant information are summarized in Table 1.

**Table 1:** Characteristics of the study population

	Family physicians (*N* = 139)	Consultants (*N* = 31)	Total (*N* = 170)
Age (years)			
Mean ± SD	43.0 ± 12.3	51.6 ± 11.1	44.6 ± 12.5
Median	39	53	43
Range	25–71	30–70	25–71
Gender [*N* (%)]			
Male	73 (52.5)	19 (61.3)	92 (54.1)
Female	66 (47.5)	12 (38.7)	78 (45.9)
Country of birth [*N* (%)]			
Israel	71 (53.4)	7 (22.6)	78 (47.6)
Other	62 (46.6)	24 (77.4)	86 (52.4)
Years in Israel (born abroad) [*N* (%)]			
Mean ± SD	24.3 ± 13.0	28.7 ± 5.3	25.6 ± 11.5
Median	25	30	27
Range	3–63	12–36	3–63
Country of graduation [*N* (%)]			
Israel	49 (36.3)	9 (29.0)	58 (34.9)
Other	86 (63.7)	22 (71.0)	108 (65.1)
Seniority (years)			
Mean ± SD	17.1 ± 12.6	26.7 ± 12.9	18.9 ± 13.2
Median	13	27	17
Range	2–44	4–48	2–48
Professional status [*N* (%)]			
Specialist	71 (51.1)	30 (96.8)	101 (59.4)
Resident	66 (47.5)	1 (3.2)	67 (39.4)
Other	2 (1.4)	0	2 (1.2)
Years of work in clinic			
Mean ± SD	11.7 ± 11.6	14.3 ± 8.1	12.2 ± 11.1
Median	6	16	9
Range	0–48	1–35	0–48
Main clinic setting [*N* (%)]			
Urban	96 (70.6)	18 (58.1)	114 (68.3)
Rural	17 (12.5)	0	17 (10.2)
Urban + rural	10 (7.4)	2 (6.5)	12 (7.2)
Community + hospital	13 (9.6)	11 (35.5)	24 (14.4)

### Physicians’ attitudes

Most physicians believed that pharmaceutical companies play an important role in promoting the practice of medicine (mean score = 3.3 ± 1.1) and that physicians should not be prohibited from meeting representatives of pharmaceutical companies (mean score = 2.2 ± 1.2). However, they also believed that interactions with representatives of pharmaceutical companies had a negative effect on the clinic workflow (mean score = 3.2 ± 1.3). Additional results appear in Table 2.

**Table 2:** Attitudes of participating physicians (1—definitely no, 5—yes, very much so)

Questions	Mean ± SD	*N*
Q1. Would you be willing to converse with representatives of pharmaceutical companies during office hours?	2.4 ± 1.2	170
Q2. Would you be willing to converse with representatives of pharmaceutical companies in your free time?	3.2 ± 1.3	170
Q3. Do you trust the information provided by representatives of pharmaceutical companies?	2.9 ± 0.8	169
Q4. Do you believe that interactions with representatives of pharmaceutical companies negatively affects the workflow in your clinic/department?	3.2 ± 1.3	169
Q5. Do you follow up on information given to you by representatives of pharmaceutical companies, using articles, Up-to-Date, or other such sites?	3.7 ± 1.2	166
Q6. Do you attend conferences subsidized by pharmaceutical companies?	3.4 ± 1.2	170
Q7. Are you willing to/do you receive gifts from representatives of pharmaceutical companies?	2.6 ± 1.3	170
Q8. Do you believe that physicians should be prohibited from meeting of representatives of pharmaceutical companies?	2.2 ± 1.2	169
Q9. Do you find the information given to you by representatives of pharmaceutical companies helpful?	3.3 ± 1.0	168
Q10. Do pharmaceutical companies play an important role in the advancement and/or practice of medicine?	3.3 ± 1.1	170
Q11. Do you believe pharmaceutical companies provide physicians with misleading information?	2.9 ± 0.9	169
Overall attitude score^a^	3.0 ± 0.6	170

^a^The overall attitude score was calculated after the score for questions 4, 5, 8, and 11 was reversed

### Comparison between the attitudes of family physicians and consultants

**Fig. 1:**
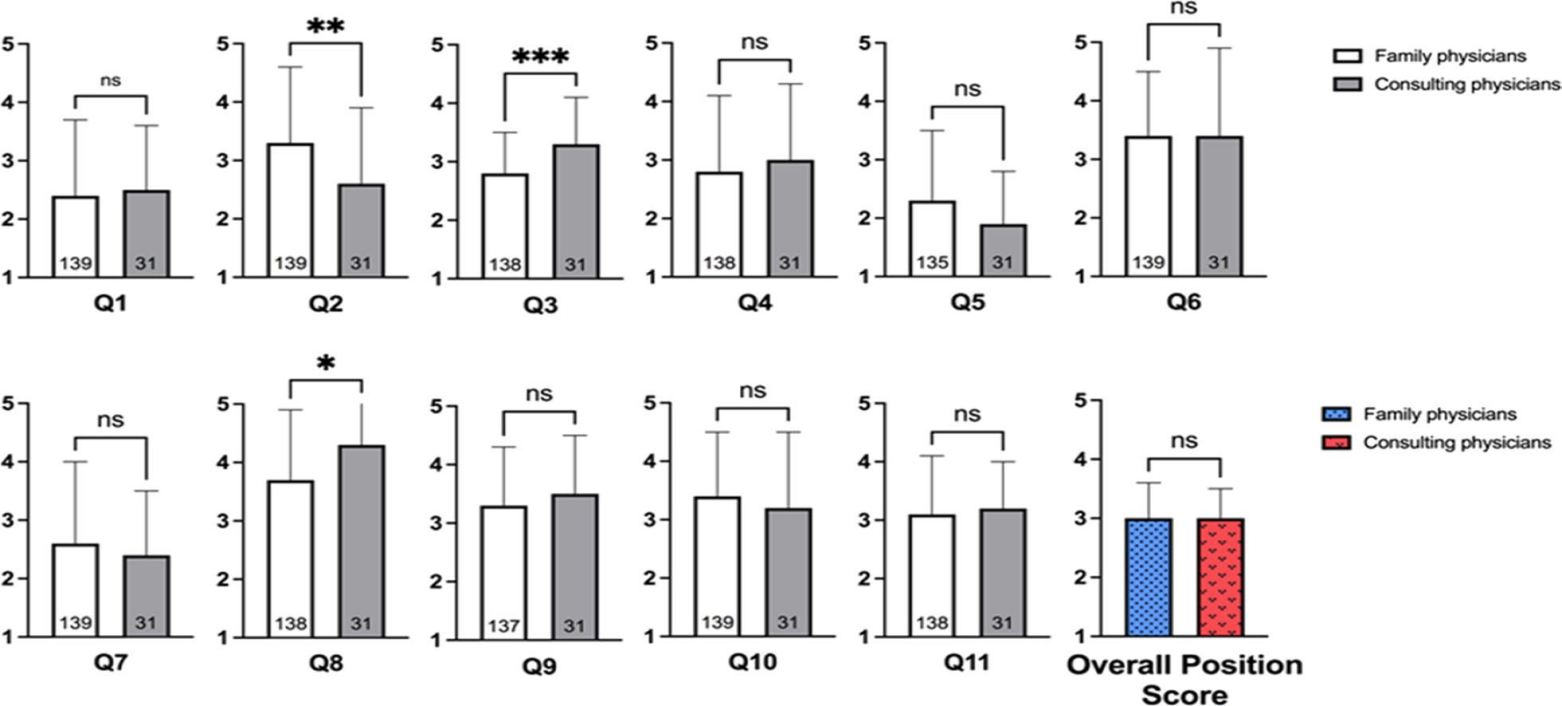
Comparison between the attitudes of family physicians and consultants. A high score indicates a high degree of agreement with the statement (1 = definitely no, 5 = yes, very much so). The overall attitude score is the mean score for all 11 questions with a higher score indicating a more positive opinion. The response order was reversed for questions 4, 5, 8, and 11 (1 = yes, very much so, 5 = definitely no). Data presented as mean ± SD. *p < 0.05, **p < 0.01 ***p < 0.001

Both groups believed that physicians should not be prohibited from meeting with representatives of pharmaceutical companies. The mean scores were 2.3 ± 1.2 and 1.7 ± 1.2 for family physicians (*n* = 138) and consultants (*n* = 31), respectively, with the consultants’ score significantly lower (*p* = 0.011, Q8). In addition, family physicians were more willing to meet with representatives of pharmaceutical companies in their free time than consultants with scores of 3.3 ± 1.3 (*n* = 139) and 2.6 ± 1.3 (*n* = 31), respectively (*p* = 0.014, Q2). There were significant differences for Q3 and Q5, as well. No significant difference was found in the overall attitude score (Fig. 1).

### Comparison between residents and specialists in family medicine

Specialists were less willing to meet with representatives of pharmaceutical companies during office hours than residents, with scores of 2.2 ± 1.2 (*n* = 101) and 2.8 ± 1.2 (*n* = 67), respectively (*p* = 0.001). Furthermore, residents thought that pharmaceutical companies played a greater role in medical progress (3.5 ± 1 (*n* = 67) than specialists (3.2 ± 1.2) (*p*-value = 0.047). No other statistically significant differences were found in other questions or in the overall attitude score.

### Comparison between male and female physicians

Our results showed two statistically significant differences between male and female physicians. First, male physicians were more willing to meet with representatives of pharmaceutical companies in their free time 3.4 ± 1.3 (*n* = 92) than females 2.9 ± 1.2 (*n* = 78) (*p* = 0.021). Second, male physicians reported that they followed up more on information given to them by representatives of pharmaceutical companies than female physicians with scores of 4.0 ± 1.1 (*n* = 89) and 3.5 ± 1.2 (*n* = 77) (*p* = 0.008). No significant difference was found in the overall attitude score.

### Comparison between graduates of medical schools in Israel and those who graduated from foreign medical schools

**Fig. 2:**
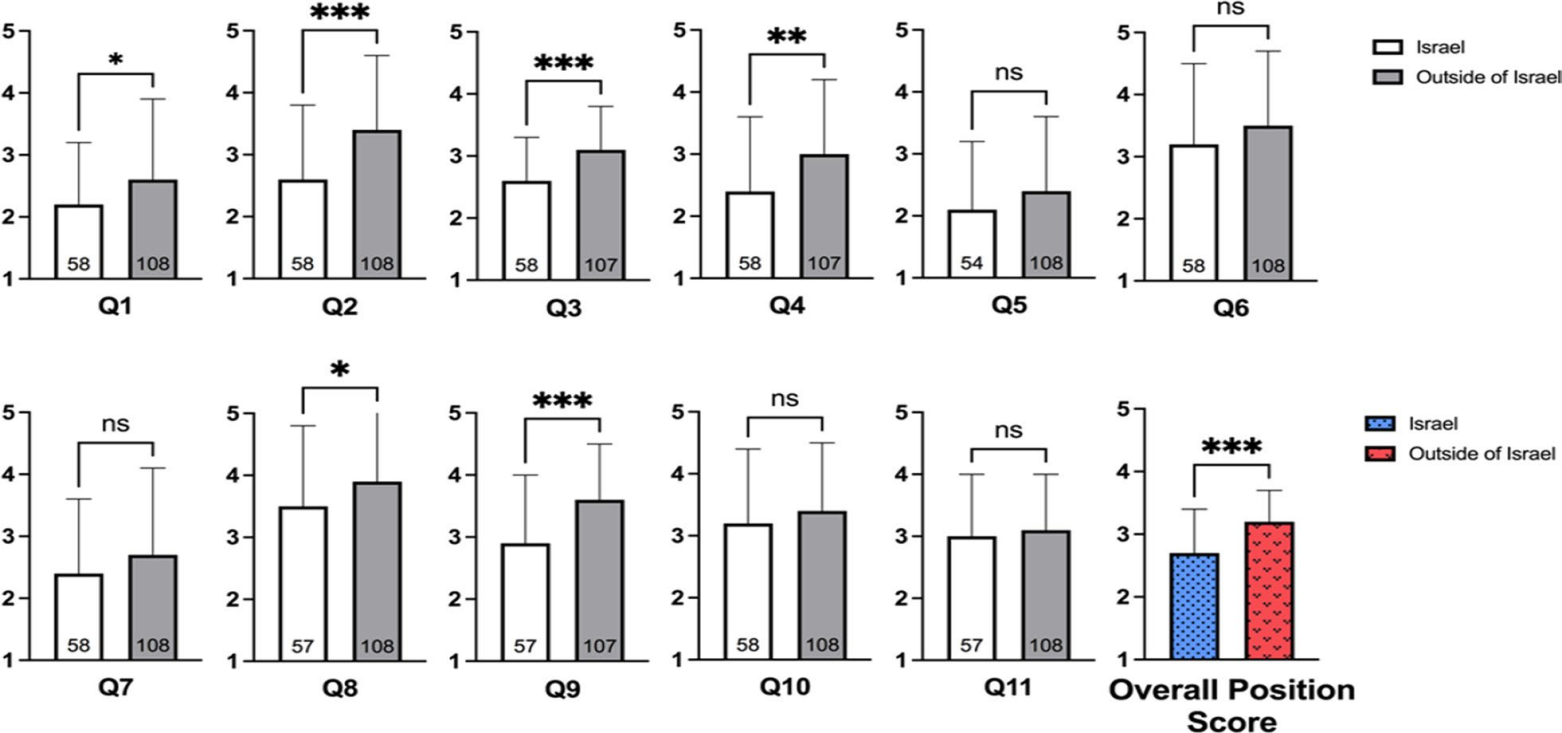
Comparison of the attitudes of physicians by country of medical school graduation. A high score indicates a high degree of agreement with the statement (1 = definitely no, 5 = yes, very much so). The overall attitude score is the mean score for all 11 questions with a higher score indicating a more positive opinion. The response order was reversed for questions 4, 5, 8, and 11 (1 = yes, very much so, 5 = definitely no). Data presented as mean ± SD. *p < 0.05, **p < 0.01 ***p < 0.001

This comparison showed significant differences in most questions, and in the overall attitude score (Fig. 2). The overall attitude scores were 2.7 ± 0.7 (*n* = 58) and 3.2 ± 0.5 (*n* = 108) for graduates from Israeli medical school and foreign medical schools, respectively (*p* < 0.0001).

Graduates of foreign medical schools were more willing to meet with representatives of pharmaceutical companies during office hours with a mean score of 2.6 ± 1.3 (*n* = 108), compared to a mean score of 2.2 ± 1 (*n* = 58), respectively (*p* = 0.035), and in their free time with a mean score of 3.4 ± 1.2 (*n* = 108) compared to 2.6 ± 1.2 (*n* = 58), respectively (*p* < 0.0001). Graduates of foreign medical schools were more opposed to the prohibition of meetings with representatives of pharmaceutical companies with a mean score of 2.1 ± 1.2 (*n* = 108) compared to 2.5 ± 1.3 (*n* = 57), respectively (*p* = 0.025). Moreover, graduates from foreign medical schools had a higher level of confidence in information provided by representatives of pharmaceutical companies with a mean score of 3.1 ± 0.7 (*n* = 107) compared to 2.6 ± 0.7 (*n* = 58) for graduates of Israeli medical schools (*p* < 0.000). In this context, graduates of foreign medical schools found the information given by representatives of pharmaceutical company’s more helpful with a mean score of 3.6 ± 0.9 (*n* = 107) compared to graduates of Israeli medical schools 2.9 ± 1.1 (*n* = 57) (*p* < 0.0001). Finally, graduates of Israeli medical schools felt more strongly that the presence of representatives of pharmaceutical companies interfered with the physician’s workflow with a mean score of 3.6 ± 1.2 (*n* = 58) compared to 3 ± 1.2 (*n* = 107) for graduates of foreign medical schools (*p* = 0.0004).

### Linear regression analysis

A linear regression model assessed factors associated with negative attitudes towards encounters with representatives of pharmaceutical companies. The model included the following variables: gender, years of work in community clinics, and country of graduation from medical school. The country of graduation had the greatest impact (beta = 0.054, sig < 0.0001). There was a strong correlation between actual years of work in the clinic and age (*r* = 0.873, *p*-value < 0.0001). This linear regression model explained 13.6% of the variance.

## Discussion

The potential consequences of interactions between physicians and representatives of pharmaceutical companies have evoked heated discussion for decades. In recent years, it has become an increasingly controversial issue in the medical community and the general population. In the past, the basis for this discussion was strongly held opinions without objective support, but the results of recent research has enabled investigators to conduct hypothesis-testing [8, 18–20].

The Covid-19 pandemic has generated an unprecedented global research effort by pharmaceutical companies and investigators to develop effective vaccines. This was coupled with an increase in related public discourse on social media platforms. A study that examined this trend found that chief among these was the discussion around vaccination use during the pandemic, which increased by 79.9%. The second most common topic was the pharmaceutical industry. The next two most discussed topics were federal health agencies such as the Centers for Disease Control and Prevention, the Food and Drug Administration, and the leaders of these health organizations, and research and clinical trials [21].

However, views expressed in the media do not necessarily reflect the attitudes of physicians towards the activity of representatives of pharmaceutical companies. In our study, we aimed to assess the attitudes of community physicians towards pharmaceutical companies and their representatives, how these activities affect physicians, physician–patient encounters, and daily work in community clinics.

The overall attitude score for all participants indicates that most participants did not have a strong opinion for or against interactions between physicians and representatives of pharmaceutical companies. A systematic review on interactions between physicians and the pharmaceutical industry found that physicians have a positive attitude towards representatives of pharmaceutical companies [22].

Most participants believed that pharmaceutical companies play an important role in advancing the practice of medicine. Most participants thought that physicians should not be prohibited from meeting representatives of pharmaceutical companies and that they would be willing to meet with representatives of pharmaceutical companies in their free time. Nevertheless, most participants believed that interactions with representatives of pharmaceutical companies had a negative effect on the clinic workflow. This apparent inconsistency can be explained by analyzing the free text answers, where many participants explained that despite the importance of this relationship, the way it is implemented today complicates management of the clinic schedule and interferes with physician–patient encounters. Some of the physicians addressed the need for better regulation. In this context, most medical and governmental institutions have installed guidelines and self-regulatory and legislative checks to regulate the relationship between physicians and the pharmaceutical industry and its representatives [23].

We performed comparisons between different subsets of community physicians and found that except for the comparison of graduates of Israeli and foreign medical schools, most did not produce significant differences. Physicians who graduated foreign medical schools viewed interactions with the representatives of pharmaceutical companies in a more positive and favorable manner. Possible explanations for this finding include differences in the culture of medical educators, in the social environment, and in the curriculum, among others [22]. A review of studies on interactions between the pharmaceutical industry and physicians in training suggested a central role for education and policy. By reviewing several models of educational interventions this review showed that different educational approaches can affect trainees in different ways [24].

This study has several limitations. First, we used a convenience sample of Israeli physicians in southern Israel, a relatively small geographic area. Moreover, the number of consultants was small due to technical difficulties that increased during the COVID-19 pandemic, delaying data collection for several months beyond the original study plan. Furthermore, the questionnaire had not been validated previously, except for pilot testing in a group of physicians, who would have been eligible to participate in the study, to test clarity and content reliability. Though a limitation, an unvalidated questionnaire was a necessary step for this study due to the scarcity of data pertaining to our research questions, and lack of validated tools. We include the questionnaire in its full form, to encourage validation in future studies. Although the response rate was high (90.9%), we cannot rule out the possibility that non-respondent bias may have influenced the results. Since this was a self-administrated questionnaire, the physicians’ responses may not fully reflect their actual opinions or behaviors, and responses are susceptible to social desirability and self-perception. Despite its limitations, we believe that this study may contribute to existing knowledge regarding interactions between physicians and pharmaceutical companies.

## Conclusions

The results of this study show that the attitudes of community-based physicians on interactions with representatives of pharmaceutical companies are mixed. On the one hand, physicians recognize the importance of these interactions for learning and staying up to date. On the other hand, they recognize that these encounters can impair the quality of medicine provided to patients.

Most participants did not hold an extreme position for or against interactions with representatives of pharmaceutical companies. Significant differences were found in the attitudes of physicians depending on whether they graduated from medical school in Israel or abroad. This suggests that education and guidance in medical school and better regulation of drug companies and their representatives could have a positive impact. Thus, these interactions should be optimized so that representatives of pharmaceutical companies could continue to contribute to physicians academically, while reducing the negative effects of these encounters.

## Supplementary Information

**Additional file 1: Table S1.** The full questionnaire (1—definitely no, 5—yes, very much so).

## Data Availability

The data that support the findings of this study are available on request from the corresponding author. The data are not publicly available due to their containing information that could compromise the privacy of research participants.
