# Supplementary material for: Community physicians’ attitudes towards meetings with representatives of pharmaceutical companies: a pilot study
Source: J Pharm Policy Pract. 2023 Jan 25;16:15. doi: 10.1186/s40545-023-00521-8 (PMC9876407; doi:10.1186/s40545-023-00521-8)
Supplement: Supplementary file 1 — Additional file 1: Table S1. The full questionnaire (1—definitely no, 5—yes, very much so). [file 40545_2023_521_MOESM1_ESM.docx]

**Additional file 1: Table S1.** The full questionnaire (1 - definitely no, 5 - yes, very much so)

| 1. Would you be willing to converse with representatives of pharmaceutical companies during office hours? | 1 | 2 | 3 | 4 | 5 |  |
| --- | --- | --- | --- | --- | --- | --- |
| 2. Would you be willing to converse with representatives of pharmaceutical companies in your free time? | 1 | 2 | 3 | 4 | 5 |  |
| 3. Do you trust the information provided by representatives of pharmaceutical companies? | 1 | 2 | 3 | 4 | 5 |  |
| 4. Do you believe that interactions with representatives of pharmaceutical companies negatively affects the workflow in your clinic/department?? | 1 | 2 | 3 | 4 | 5 |  |
| 5. Do you follow up on information given to you by representatives of pharmaceutical companies, using articles, Up-to-Date, or other such sites? | 1 | 2 | 3 | 4 | 5 |  |
| 6. Do you attend conferences subsidized by pharmaceutical companies? | 1 | 2 | 3 | 4 | 5 |  |
| 7. Are you willing to / do you receive gifts from representatives of pharmaceutical companies? | 1 | 2 | 3 | 4 | 5 |  |
| 8. Do you believe that physicians should be prohibited from meeting of representatives of pharmaceutical companies? | 1 | 2 | 3 | 4 | 5 |  |
| 9. Do you find the information given to you by representatives of pharmaceutical companies helpful? | 1 | 2 | 3 | 4 | 5 |  |
| 10. Do pharmaceutical companies play an important role in the advancement and/or practice of medicine? | 1 | 2 | 3 | 4 | 5 |  |
| 11. Do you believe pharmaceutical companies provide physicians with misleading information? | 1 | 2 | 3 | 4 | 5 |  |
